# The reliability and minimal detectable change of Timed Up and Go test in individuals with grade 1 – 3 knee osteoarthritis

**DOI:** 10.1186/s12891-015-0637-8

**Published:** 2015-07-30

**Authors:** Ahmad Alghadir, Shahnawaz Anwer, Jean-Michel Brismée

**Affiliations:** Rehabilitation Research Chair, College of Applied Medical Sciences, King Saud University, Riyadh, Saudi Arabia; Dr. D. Y. Patil College of Physiotherapy, Dr. D. Y. Patil Vidyapeeth, Pune, India; Center for Rehabilitation Research, Texas Tech University Health Sciences Center, Lubbock, Texas USA

## Abstract

**Background:**

The Timed Up and Go (TUG) test is quick and easy tests to assess patients’ functional mobility. However, its reliability in individuals with knee osteoarthritis (OA) has not been well established. The aims of this study were to determine the reliability and minimal detectable change of the TUG test in individuals with doubtful to moderate (Grade 1–3) knee OA.

**Methods:**

Sixty-five subjects (25 male, 40 female), aged 45–70 years, with knee OA participated. Inter-rater reliability was assessed using two observers at different times of the same day in an alternating order. Intra-rater reliability was assessed on two consecutive visits with a 2-day interval. The standard error of measurement (SEM) and the minimum detectable change (MDC) were calculated to determine statistically meaningful changes.

**Results:**

Intra-rater and inter-rater reliability were 0.97 (95 % confidence interval [CI], 0.95 – 0.98) and 0.96 (95 % confidence interval [CI], 0.94 – 0.97), respectively. The MDC, based on measurements by a single rater and between raters, was 1.10 and 1.14 seconds, respectively.

**Conclusions:**

The TUG is a reliable test with adequate MDC for clinical use in individuals with doubtful to moderate knee OA.

## Background

Knee osteoarthritis (OA) is a common musculoskeletal disorder affecting the functional mobility of older individuals [1, 2]. The evidence of radiographic knee OA has been reported to be 53.3 % in male and 60.9 % in female adults aged 30 – 93 years in the Middle East [3]. Knee OA is likely to become the eighth most important global cause of disability in men and the fourth most important in women [4]. The presence of knee pain, decreased functional mobility, stiffness and reduced quadriceps strength has been associated with knee OA and may lead to physical disability [5–7]. Because a major aim of rehabilitation programs for knee OA is to optimize patients’ functional mobility to carry out their activities of daily living (ADLs), therapist require a valid and reliable tool to assess patients’ functional mobility at baseline and post intervention.

The Timed Up and Go (TUG) test is one of the simple and quick tests to assess patients’ functional mobility. Podsiadlo and Richardson [8] recorded the time taken to complete the TUG in a group of frail elderly subjects with stroke, Parkinson’s disease, arthritis, cerebellar disorders, or general deconditioning. In addition to excellent reliability (ICC 0.99), the TUG scores shown moderate correlation with the Barthel Index (*r −*0.51), gait speed (*r −*0.55), and the Berg Balance Scale score (*r −*0.72) [8]. Several other studies reported good test-retest reliability of TUG test in specific subject populations, including community-dwelling older adults [9, 10], individuals with Parkinson’s disease [11, 12], and unilateral lower-limb amputation [13]. Previous research suggested that the TUG test had the capacity, in community dwelling people, to predict the patient's ability to go outside alone safely and to function in other settings [8]. Yeung et al. [14] investigated the test – retest reliability of TUG test in a group of patients admitted in inpatient orthopaedic ward (most of them had either total hip or total knee arthroplasty surgery). They reported moderate test – retest reliability (ICC 0.80) and concluded that the TUG test was reliable and valid to assess group changes of patients in orthopaedic rehabilitation wards.

Recently, the Osteoarthritis Research Society International (OARSI) recommended a set of five performance-based tests of physical function, including the TUG test in individuals diagnosed with hip or knee OA [15]. The authors recommended TUG test because it demonstrated good measurement properties in people with OA and other populations [16–20]. In addition, Dobson et al. [21] conducted a systematic review on the measurement properties of performance-based measures to assess physical function in hip and knee OA. They reported that sit to stand tests with the best measurement evidence included the TUG test and the 30-second chair stand test for hip/knee OA. In a previous study, Norén et al. [22] investigated the applicability and reliability of some balance assessment methods, including the TUG test, in individuals with peripheral arthritis. They reported that the individuals with severe disability were generally able to perform the TUG test.

Although Kennedy et al. [16] investigated measurement properties of four performance measures including TUG test in patients with advanced OA undergoing total hip or knee arthroplasty, no study to date has estimated the reliability and minimal detectable change of TUG test in a population with doubtful to moderate (Grade 1–3) knee OA. Hence, the purpose of this study was to estimate the reliability and MDC of TUG test in individuals with doubtful to moderate knee OA.

## Methods

### Participants and criteria

All patients diagnosed with knee OA (unilateral or bilateral) by the Physician as per the American College of Rheumatology (ACR) [23] who were referred for outpatient physiotherapy were invited to participate after explanation of the study. Subjects agreeing to participate then signed written consent. Both male and female in the age range of 45 – 70 years with pain in and around the knee and radiological evidence of primary grade 1 – 3 knee OA on the Kellgren and Lawrence scale [24] were included. Subjects with grade 4 knee OA as per Kellgren and Lawrence scale were excluded as well as subjects with any central or peripheral nervous system involvement, a history of a systemic arthritic condition or of knee surgery to either knee within the past three months. The Kellgren and Lawrence scale classifies OA into four grades as follows: Grade 0 indicates no radiographic findings of OA; Grade 1 indicates minimal osteophytes of doubtful clinical significance; Grade 2 indicates definite osteophytes with unaffected joint space; Grade 3 indicates definite osteophytes with moderate joint space narrowing; and Grade 4 indicates definite osteophytes with severe joint space narrowing and subchondral sclerosis [24]. The Institutional Research Ethics Committee of the Rehabilitation Research Chair, King Saud University, Riyadh, Saudi Arabia, approved the study.

### Procedures of data collection

The study participants’ age, sex, height, weight, body mass index (BMI), pain, function, and grade of knee OA were recorded. Pain intensity and knee function were measured using the numerical rating scale (NRS) and the reduced Western Ontario and McMaster Universities Osteoarthritis (WOMAC) index, respectively. The NRS consists of an 11 point horizontal scale from 0 to 10, with 0 meaning no pain at all and 10 describing the worst pain ever. It is a reliable and valid instrument for assessing musculoskeletal and arthritic pain [25, 26]. The 5-point likert version of the reduced WOMAC index was used to assess knee function [27, 28].

Two licensed physiotherapists with more than 8 years of clinical practice and experience in the TUG test administration performed inter-rater reliability testing at different times of the same day in an alternating order. Both clinicians were trained in the administration of TUG test for the purpose of standardization of the instructions. For intra-rater reliability testing, the same physiotherapists performed the TUG on two consecutive visits with 2-day interval. The TUG test was administered by one examiner in a quiet area [8]. Subjects were instructed to stand up from the chair, walk 3 meters comfortably and safely, come back and sit back in the chair. The time taken to complete this task was measured with a stopwatch timed to the nearest 1/100 seconds. A practice trial was given and then followed by 2 recorded trials. An average of the 2 recorded trials was used in data analysis.

### Statistical analysis

Descriptive statistics were used to analyze subjects’ demographic characteristics and baseline measurements. To determine inter- and intra-rater reliability of TUG measurements between the 2 testing sessions, intraclass correlation coefficients (ICC_2,1_) were used. The Bland-Altman plot method was then used to assess the agreement between two readings. The plot comprises of the average of the paired values from two readings on the x-axis and the difference of each set of readings on the y-axis. Data were visually interpreted to determine the consistency of two scores. The standard error of measurement (SEM) and the minimum detectable change (MDC) were calculated using the results of the reliability analyses. The SEM is the commonest statistic reported in previous studies for assessing statistically meaningful changes of a health outcome [29, 30]. MDC was calculated as 1.96√2 (SEM) [31]. All statistical analyses were performed with SPSS for Windows version 22 (Statistical package for Social Sciences, IBM Inc.), and the significance level was set at 0.05.

## Results

Of the 80 subjects recruited, 15 (Grade 4 OA, n = 8; age >80 years, n = 7; men 5, women 10) were excluded due to not meeting inclusion criteria. Table 1 details the participants’ characteristics. The mean age and standard deviation of the male and female participants were 54.3 (10.1) and 51.4 (9.7) years, respectively. Thirty-nine participants (40 %) had unilateral while the others (60 %) had bilateral knee OA. Thirty-eight participants had grade 1, 12 grade 2, and 15 grade 3 knee OA as per Kellgren and Lawrence grading system. Table 2 details the baseline scores of TUG, NRS, and WOMAC.

**Table 1:** Participant’s characteristics

Gender, No. (%)	
Male	25 (38.5)
Female	40 (61.5)
Age, years	
Mean (SD)	54.87 (9.87)
Minimum – maximum	45 – 70
Height, meter (m)	
Mean (SD)	1.69 (0.06)
Minimum – maximum	1.56 – 1.86
Weight, Kg.	
Mean (SD)	83.15 (12.91)
Minimum – maximum	56 – 112
BMI, Kg./m^2^	
Mean (SD)	29.04 (3.72)
Minimum – maximum	21.38 – 38.75
K/L rating score, no. (%)	
Grade 1	38 (58.5)
Grade 2	12 (18.5)
Grade 3	15 (23)
Limb involvement, no. (%)	
Unilateral	39 (60)
Bilateral	26 (40)

*BMI* Body mass index, *K/L* Kellgren and Lawrence scale

**Table 2:** Descriptive statistics of baseline scores

TUG score (Seconds), All participants	
Mean (SD)	10.88 (3.62)
Minimum – maximum	6.40 – 19.90
TUG score (Seconds), Male	
Mean (SD)	10.18 (2.95)
Minimum – maximum	7.05 – 18.76
TUG score (Seconds), Female	
Mean (SD)	11.32 (3.96)
Minimum – maximum	6.40 – 19.90
TUG score (Seconds), Doubtful knee OA (Grade 1)^a^	
Mean (SD)	8.46 (0.79)
Minimum – maximum	6.40 – 9.85
TUG score (Seconds), Definite knee OA (Grade 2/3)^a^	
Mean (SD)	14.29 (3.28)
Minimum – maximum	10 – 19.90
NRS (0–10)	
Mean (SD)	5.00 (2.17)
Minimum – maximum	1 – 9
WOMAC total score (0–48)	
Mean (SD)	16.05 (8.91)
Minimum – maximum	3 – 35

*OA* Osteoarthritis, *TUG* Timed up and go test, *NRS* Numerical rating scale, *WOMAC* Western Ontario and McMaster Universities Osteoarthritis index

^a^Grading as per Kellgren and Lawrence scale [24]

### Intra- and inter-rater reliability

**Fig. 1:**
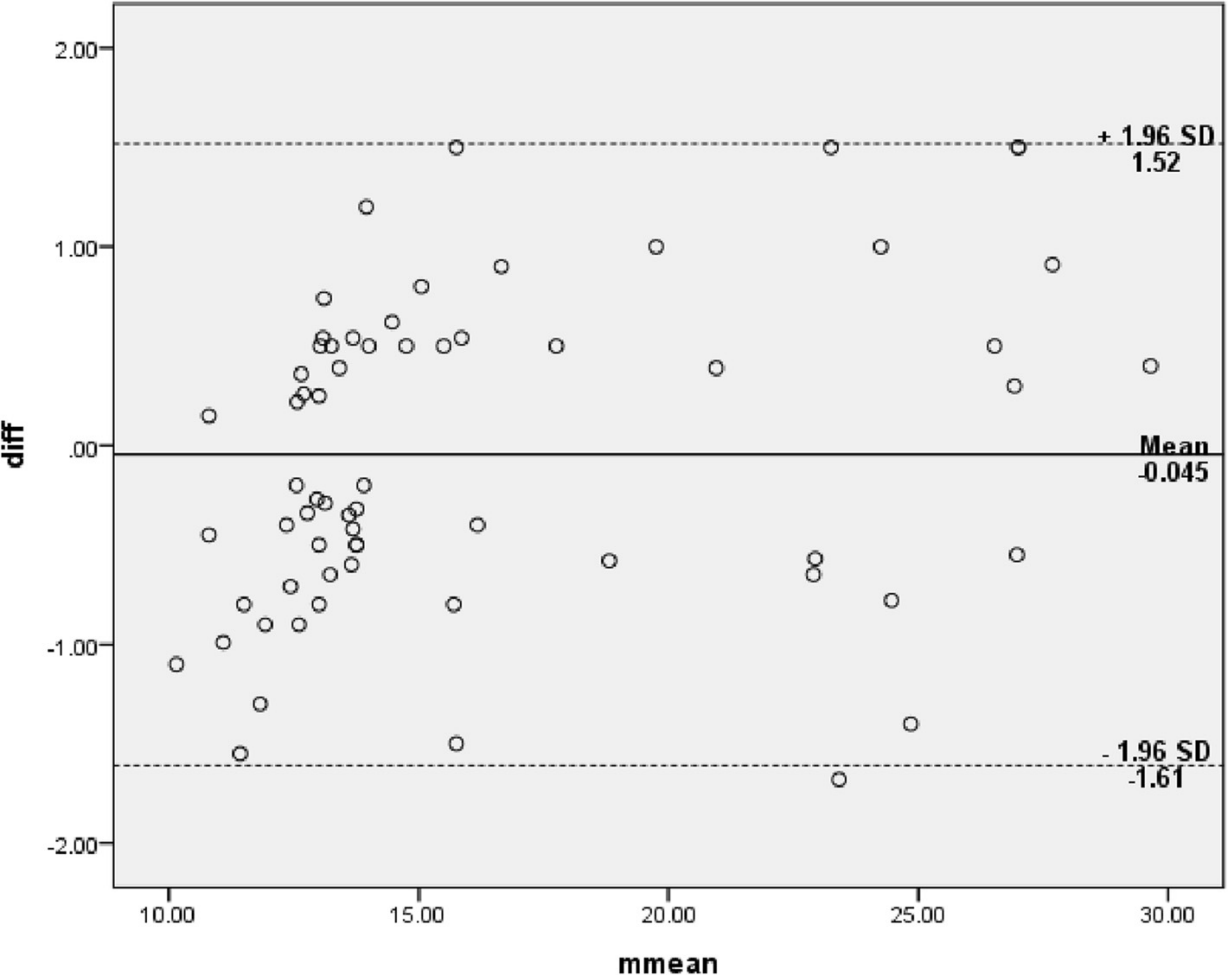
Bland-Altman plot of difference of two raters with mean (bias) and ± standard deviation (SD) of differences of two raters

**Fig. 2:**
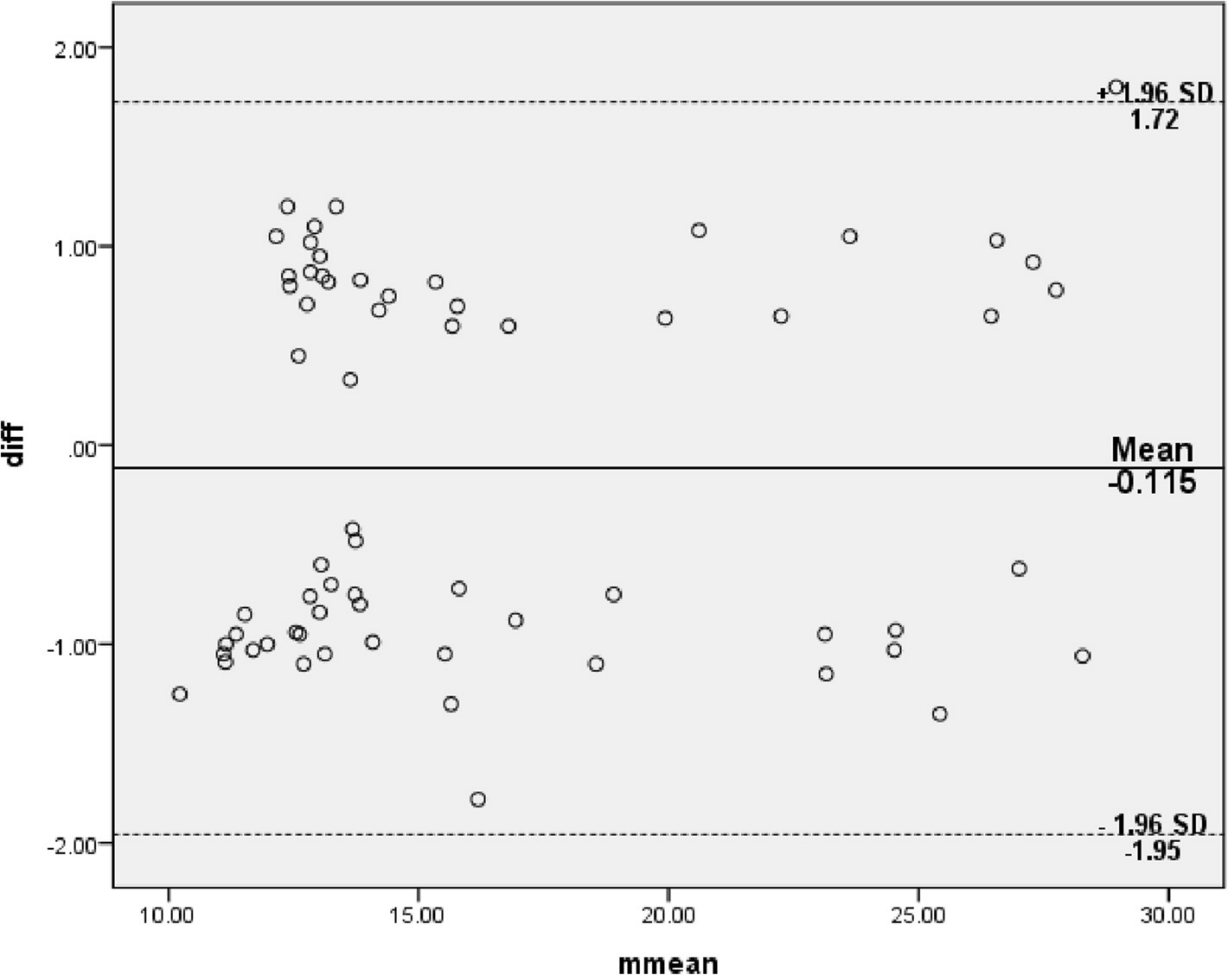
Bland-Altman plot of difference of two readings for the same rater with mean (bias) and ± standard deviation (SD) of differences of two readings

The TUG test for all the participants showed excellent intra- and inter-rater reliability (ICC .97 and .96, respectively) (Table 3). The Bland-Altman limits of agreement depicted in Figs. 1 and 2 showed a reasonable agreement between the 2 raters (inter-rater) and good agreement between two readings (intra-rater) when differences between the two readings were plotted against the mean of two readings for all scores. Table 4 shows gender- and grade (OA severity)-wise intra- and inter-rater reliability of the TUG test. The TUG test for male participants showed excellent intra- and inter-rater reliability (ICC .98 and .97, respectively). The TUG test for female participants showed excellent intra- and inter-rater reliability (ICC .98). The TUG test for doubtful knee OA (Grade 1) showed good intra- and inter-rater reliability (ICC .73 and .71, respectively). The TUG test for definite knee OA (Grade 2–3) showed excellent intra- and inter-rater reliability (ICC .97 and .97, respectively).

**Table 3:** Inter- and Intra-rater reliability of Timed Up and Go test – ICCs, SEM, MDC and Bland and Altman tests

	ICC, SEM and MDC			Bland and Altman test			
	ICC (95 % CI)	SEM	MDC	đ	95 % CI for đ	SD_diff_	95 % limit of agreement
Inter-rater reliability	0.96 (0.94 – 0.97)	0.17	1.14	−.045	−.24 – .15	.80	−1.61 – 1.52
Intra-rater reliability	0.97 (0.95 – 0.98)	0.16	1.10	−.115	−.34 – .11	.94	−1.95 – 1.72

*ICC* intraclass correlation coefficients, *SEM* standard error of measurement, *MDC* minimal detectable change, *đ* mean difference, *95 % CI for đ* 95 % confidence interval for the mean difference, *SD*_*diff*_ standard deviation of the differences

**Table 4:** Inter- and Intra-rater reliability of Timed Up and Go test: gender- and grade (OA severity)-wise

	Male ICC (95 % CI) N = 25	Female ICC (95 % CI) N = 40	^a^Doubtful OA (Grade 1) ICC (95 % CI) N = 38	^a^Definite OA (Grade 2–3) ICC (95 % CI) N = 27
Inter-rater reliability	0.97 (0.93 – 0.98)	0.98 (0.97 – 0.99)	0.71 (0.44 – 0.85)	0.97 (0.94 – 0.98)
Intra-rater reliability	0.98 (0.95 – 0.99)	0.98 (0.97 – 0.99)	0.73 (0.48 – 0.85)	0.97 (0.95 – 0.99)

*OA* Osteoarthritis

^a^Grading as per Kellgren and Lawrence scale [24]

### Measurement error and minimum detectable change

The SEM values were 0.17 seconds and 0.16 seconds, based on repeated measurements for inter- and intra-rater, respectively. The MDCs based on the SEM for inter- and intra-rater were 1.14 and 1.10 seconds, respectively (Table 3).

## Discussion

Recently, the Osteoarthritis Research Society International (OARSI) recommended the use of the TUG test as a performance-based test of physical function in individuals diagnosed with hip or knee OA [15]. In addition, Dobson et al. [21] reported that the TUG test displayed best measurement evidence among sit to stand tests for hip/knee OA. This is the first study to estimate the reliability and MDC of TUG test in individuals with doubtful to definite radiographic knee OA (Kellgren and Lawrence grades 1–3). The results indicated that the TUG test is sufficiently reliable and sensitive to detect small clinical changes, with psychometric properties in agreement with those reported in most studies on the elderly population (ICC range, 0.92–0.99) [8, 10–13]. Both men and women displayed excellent reliability (ICC range, 0.97 – 0.98). Similarly, Norén et al. [22] reported excellent reliability (r = 0.97) of TUG test in individuals with peripheral arthritis. However, the subjects in their study were primarily individuals with rheumatoid arthritis.

In the present study, the participants with doubtful and definite knee OA had good and excellent reliability (ICC .71 and .97, respectively). Likewise, Kennedy et al. [16] reported moderate to good reliability of the TUG in patients with advanced OA undergoing total hip or knee arthroplasty. Although the characteristics of the participants included in the Kennedy et al. study [16] and in the present study were different in regard to the severity of the OA condition, both studies found good reliability, thereby indicating the value of the TUG for populations with various levels of OA severity. Patients with advanced knee OA would be expected to display increased performance variability, thus reducing the reliability of repeated measurements.

The mean TUG score obtained in among individuals with knee OA (10.9 ± 3.6 s) was lower than that of older adults who functioned independently (8.1 ± 1.3 s) [32]. The female participants had lower TUG score than male participants (11.3 s versus 10.2 s). Similarly, the participants with definite knee OA had lower TUG score than doubtful knee OA (14.3 s versus 8.5 s). The presence of knee pain and quadriceps muscle weakness is associated with knee OA [5–7], which could explain the lower TUG score in subjects with knee OA as compared to healthy older adults.

The values of SEM and the MDC were used to calculate measurement error. It is the speculative difference between an observed score on any specific assessment and the actual score for the method [33]. The value of the SEM and MDC provides a threshold for interpreting the TUG over time. The difference between the MDC values based on the SEM for one rater (1.10 s) and 2 raters (1.14 s) was small (0.04 s); therefore, we suggest choosing 1 MDC value to avoid the use of multiple values. Hence, we chose to use the larger MDC (that based on the SEM between two raters). Using this criterion, when the TUG score changes by over 1.14 s, one can be reasonably sure that a true change has occurred, and not just measurement error or noise. Knowledge of the MDC is important to compare the changes in performance-based measures of function in individuals with knee OA. However, Kennedy et al. [16] reported higher SEM (1.07 s) and MDC (2.49 s) as compared to the present study. This may be due to the difference in the participants’ characteristics. Participants in their study were individuals with advanced OA undergoing total hip or knee arthroplasty, while individuals with grade 4 knee OA were excluded from this study. In addition, Norén et al. [22] reported higher SEM (1 s) for individuals with peripheral arthritis with mild to severe disability.

The radiographic knee OA severity for various grades is considered debatable in the literature and the use of radiographs for diagnosing knee OA in person with knee pain in primary care is considered inappropriate [34–36]. Hence, we opted to keep the phrase “doubtful to moderate knee osteoarthritis” for grade 1–3.

Generalization of our results should be limited to the individuals with knee OA with a radiographic grade up to 3 as per Kellgren and Lawrence scale [24]. The sample did not include individuals with grade 4 knee OA. The presence of grade 4 knee OA would be expected to increase variability of performance, thus reducing the reliability of repeated measurements. In addition, inclusion of healthy control group could have improved the validity. Despite these limitations, we believe that our study provides estimates of reliability and MDC of TUG scores in individuals with doubtful to moderate knee OA, warranting replication by clinicians in other countries using larger samples of subjects. It would be interesting for future studies to examine the effect of treatment on TUG scores, pain and functional mobility in individuals with knee OA.

## Conclusions

The intra- and inter-rater reliability of the TUG measurements were good to excellent with adequate MDC for clinical use in individuals with doubtful to moderate (Grade 1–3) knee OA. Further study is warranted to validate the TUG test as a single measure of physical function of individuals with knee OA.

## Abbreviations

TUG: Timed Up and Go
MDC: Minimal detectable change
OA: Osteoarthritis
NRS: Numerical rating scale
WOMAC: Western Ontario and McMaster Universities Osteoarthritis Index
